# Occurrence of BVDV Infection and the Presence of Potential Risk Factors in Dairy Cattle Herds in Poland

**DOI:** 10.3390/ani10020230

**Published:** 2020-01-31

**Authors:** Krzysztof Rypuła, Katarzyna Płoneczka-Janeczko, Michał Czopowicz, Malgorzata D. Klimowicz-Bodys, Sergey Shabunin, Georges Siegwalt

**Affiliations:** 1 Division of Infectious Diseases of Animals and Veterinary Administration, Department of Epizootiology and Clinic of Birds and Exotic Animals, Faculty of Veterinary Medicine, Wroclaw University of Environmental and Life Sciences, pl. Grunwaldzki 45, 50-366 Wroclaw, Poland; katarzyna.ploneczka-janeczko@upwr.edu.pl (K.P.-J.); malgorzata.klimowicz-bodys@upwr.edu.pl (M.D.K.-B.); 2 Division of Veterinary Epidemiology and Economics, Institute of Veterinary Medicine, Warsaw University of Life Sciences, Nowoursynowska Street 159c, 02-776 Warsaw, Poland; mczopowicz@gmail.com; 3 All-Russian Veterinary Research Institute of Pathology, Pharmacology and Therapy, Lomonosova Street 114 b, 394087 Voronezh, Russia; main@vsau.ru; 4 Boehringer Ingelheim RCV GmbH & Co KG, Animal Health, Dr. Boehringer-Gasse 5-11, 1121 Wien, Austria; georges.siegwalt@boehringer-ingelheim.com

**Keywords:** BVDV, dairy cattle, risk factors

## Abstract

**Simple Summary:**

The bovine viral diarrhea virus (BVDV) causes one of the most common and economically important viral diseases of cattle. It affected cattle reproductive disorders in breeding cattle as well as decreased productivity through increased forced culling, morbidity, and mortality, all of which can be observed on the herd level. The aim of our study was the estimation of the occurrence of BVDV infection in different regions of Poland and the analysis of the different factors that could be correlated with the productive results. We evaluated 354 cattle herds. The presence of antibodies against the BVD virus was found in 33.3% of examined herds, and the heterogenous distribution of BVDV-positive herds in all regions of Poland was confirmed. We found that the rate of BVDV infection was strongly correlated with the geographical location of the examined cattle populations in Poland, the grazing of the animals, and the purchasing of new animals to be introduced to herds.

**Abstract:**

The aim of the study was to analyze the risk factors of BVDV infection, in different regions of Poland, with respect to certain parameters of animal health, including productivity, herd management practices, the presence of BVDV, and the effect of non-vaccination. A total of 354 cattle herds were estimated and linked to the analysis of the different factors that might be correlated with productive results. The presence of antibodies against BVDV was found in 33.3% of examined herds, and the heterogenous distribution of BVDV-positive herds in all regions of Poland (*p* = 0.001) was confirmed. We found statistical correlations between BVDV infection and pasture (*p* = 0.004) and the number of grazing animals (*p* < 0.001), and also the purchase of animals for replacement (*p* = 0.004) was observed. Production or clear clinical outcomes potentially linked to BVDV infection in the herd have not been observed. The results of this study indicate that the rate of BVDV infection was most strongly correlated with the geographical location of the examined cattle populations in Poland. The second most significant factors were the grazing of animals and the purchasing of new animals to be introduced to herds. The strengthened procedures of management, as well as the implementation of eradication programs, should be considered in the examined herds.

## 1. Introduction

The bovine viral diarrhea virus (BVDV) is a small single-stranded positive-sense RNA virus, classified in the genus Pestivirus within the Flaviviridae family [1]. Two species, BVDV-1 and BVDV-2, are discriminated on the basis of genetic and antigenic differences. The International Committee on Taxonomy of Viruses (ICTV) recognizes four main separate species that are members of the Pestivirus genus: BVDV- 1, BVDV-2, border disease virus (BDV), and classical swine fever virus (CSFV) [2]. Moreover, a growing number of tentative Pestivirus species isolated from domestic and wild ruminant species and from pigs, (“Giraffe”, “Pronghorn” or “Bungowannah” viruses) [3,4,5] as well as atypical (Th/04_KhonKaen, HoBi-like, and CH-KaHo/cont) Pestivirus species, has been identified and described [6,7,8,9]. 

This virus causes one of the most common and economically important viral diseases of cattle. It affects industrialized cattle farming systems by inducing reproductive disorders (abortion, overdue birth—prolonged gestation, reduced fertility) in breeding cattle and by reducing herd productivity through increased forced culling, morbidity, and mortality [10,11]. 

The principal source of infection is a persistently infected (PI) animal. Healthy, immunocompetent adult cattle and calves tend to suffer from acute BVDV infections, causing seroconversion that occurs 2–3 weeks after infection, leading to lifelong immunity. The ability of BVDV to cross the placenta and fetal infection during early pregnancy can result in the birth of PI calves [12,13]. Yet, transiently infected animals (TI) are considered to be of minor importance in the spread of the disease [13]. However, BVDV due to TI animals might circulate within a herd for a prolonged period, even in the absence of PIs [14]. Most control programs consist of the identification and elimination of PI animals (removing the source of infection TI animals) based on this pathogenesis [15]. The aim of this study was to analyze the risk factors of BVDV infection in different regions of Poland with respect to certain parameters of animal health, including productivity, herd management practices, the presence of BVDV, and the effect of non-vaccination. 

## 2. Materials and Methods 

### 2.1. Sample Collection

**Figure 1:**
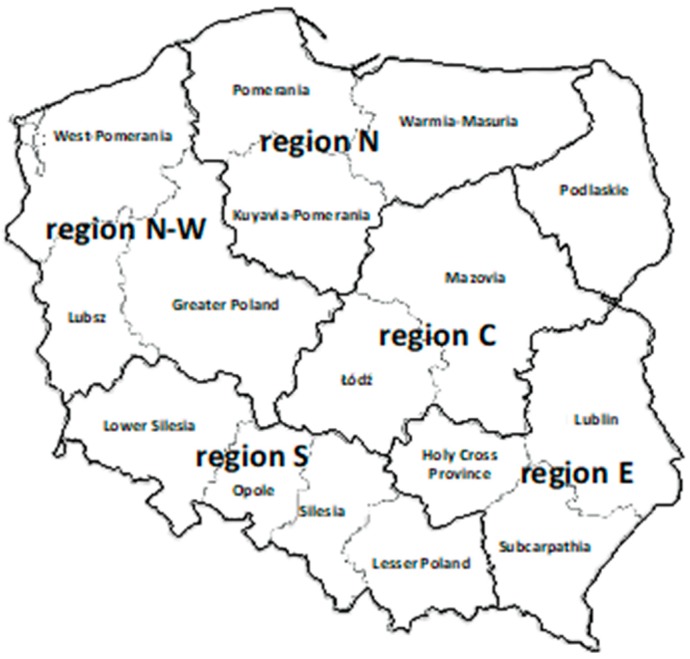
Examined geographical regions of Poland according to the Chief Statistical Office with modification, including the concentration of cows per 100 ha of the agricultural area.

Samples were collected from cattle herds where the routine laboratory milk tests have been performed to estimate the current status of the targeted infectious diseases. In total, BTM (bulk tank milk) samples were collected at 354 dairy farms from five regions of Poland between January 2015 and May 2016. These five geographical regions included: Central (C), consisting of Mazovia and Łódź Voivodships; Southern (S), consisting of Lower Silesia, Opole, Silesia, and Lesser Poland Voivodships; North-Western (N-W), consisting of Greater Poland, Lubsz, and West-Pomerania Voivodship; Northern (N), consisting of Warmia-Masuria, Kuyavia-Pomerania, and Pomerania Voivodships; Eastern (E), consisting of Holy Cross Province, Subcarpathia, Lublin, Podlaskie Voivodships (Figure 1). These regions were designated according to the Chief of Statistical Office, taking into account the number of cows per 100 ha of agricultural area [16]. Thus, we investigated: (C) 41 herds, (S) 59 herds, (N-W) 98 herds, (N) 93 herds, and (E) 63 herds. The concentration of cows per 100 ha of the agricultural area in the selected regions were: (C) Mazovia—25.5 and Łodz—17.8; (S) Lower Silesia—4.4, Opole—8.6, Silesia—11.9, and Lesser Poland—13.5; (N-W) Greater Poland—15.7, Lubsz—7.7 and West-Pomerania—4.7; (N) Warmia-Masuria—22.0, Kuyavia-Pomerania—14.5 and Pomerania—9.3; (E)—Holy Cross Province—11.2, Subcarpathia—11.2, Lublin—9.7, and Podlaskie—41.9. None of the cattle in any of the farms had been vaccinated against BVDV over the preceding year, and no other special criteria were included when selecting the farms. 

BTM samples were collected into 100 ml containers with no preservatives. The containers were labelled in detail by appointed veterinarians. Collected samples were transported directly to the Diagnostic Laboratory EPI-VET, the Faculty of Veterinary Medicine, and Wroclaw, which implements a quality management system (ISO/IEC 17025:2005 + API:2007 + AC:2007). All samples were transported to the laboratory at 4–8 °C within 24 h after collection. BTM samples were centrifuged at 1000× *g* for 15 min at 4 °C. Fat was skimmed off and finally the supernatant was collected. Centrifugation was repeated twice to ensure this. Then, the supernatant was stored in 1 mL aliquots at −20 °C, until analysis.

### 2.2. ELISA Test

To investigate and detect antibodies of NS-3 BVDV an indirect ELISA test was used (Bio-X Diagnostics, Rocheford, Belgium). The obtained results were expressed as S/P. The relative amount of antibodies was calculated using the positive control as a reference, according to the manufacturer’s instructions. The relationship is expressed as a s/p ratio (Sample to Positive Ratio).

val ≤ 20% = 0 negative;
20% < val ≤ 40% = (+);
40% < val ≤ 60% = (++);
60% < val ≤ 80% = (+++);
80% < val ≤ 100% = (++++);
100% < val = (+++++).

A sample was considered positive if the obtained result was greater than or equal to one plus (+).

### 2.3. Questionnaire

To analyze how BVDV infection affects production in cattle, veterinarians who worked at the selected farms (with the cooperation of the animal owners) were asked to complete questionnaires. The questionnaires contained general and specific information, including: the total number of animals in the herd, the number of animals in lactation, the average age at first calving, the average calving interval, milk yield/production, and access to the pasture of particular age groups. Information about the status of animal health was also given, including: diarrhea in calves, symptoms of respiratory diseases in calves/heifers, abortions, and fetopathies. The percentage of cows with clinical mastitis (which occurred in the last two months before this study) was recorded, along with data on the number of somatic cells in the milk. The persons responsible for the herds were consulted on what they considered to be the main causes of slaughtered animals in the herd, as well as fertility problems, mastitis, lameness, and information about prophylaxis against BVDV carried out in herds and in the newly-purchased animals (those purchased within the last year).

### 2.4. Statistical Analysis

Numerical variables were presented as the median and interquartile range (IQR), and categorical variables were presented as the counts and percentages of herds with respect to each category. Between-group comparisons of nominal and ordinal variables were performed with the Pearson’s chi-square test and the Mann–Whitney U test, respectively. Ninety five percent confidence intervals (CI 95%) for proportions were calculated using the Wilson score method. All statistical tests were two-sided. A significance level (α) was set at 0.05. Analysis was performed in TIBCO Statistica 13.3 (TIBCO Software Inc., Palo Alto, CA, USA). Bar charts were prepared in Microsoft Office Excel®.

### 2.5. Ethics Statement

The BTM used in this study originally came from the samples used as a material for a routine diagnostic of the health status of dairy cattle herds. The research outline was submitted to the II Local Ethics Committee for Animal Experiments in Wroclaw. Due to the non-invasive procedure of the samples collection, the Ethics Committee qualified the study as research that did not require ethics committee approval.

## 3. Results

The study was conducted in 354 dairy cattle herds, with herd size ranging from 30 to 831 heads of cattle. The presence of antibodies against BVDV were found in 118 herds, giving the herd-level seroprevalence of BVDV in Poland of 33.3% (CI 95%: 28.6%, 38.4%). Table 1 shows the number and percentage of positive herds in relation to the relative amount of antibodies, which were expressed as s/p. 

A high percentage (46.2%) of seropositive herds was detected in the (N) region, which is characterized by a high concentration of cattle counted per 100 ha of agricultural area. In this region, a typical feature is a large number of the relatively small herds, which restricts access to the pastures. However, the grazing of animals in small herds leads to more frequent contact with infected animals as well as contaminated surfaces/equipment. Additionally, the feeding method, which is closely related to the transport and distribution of the feed given to the animals, differed among the (N), (C), and (N-W) regions. This phenomenon is reflected in the proportion of grass grazed in the diet.

An interesting observation of our study was that a greater frequency of BVDV infection was detected in herds where the owners had not purchased animals in the last year. Animals were introduced into stocks in the years 1995–2005 as a commonly used strategy to maintain the genetic quality of livestock, including milk yields in this period of time [17]. Additionally, the low percentage of seropositive herds in the (N-W) region, where vaccinations were not implemented, was correlated to the period before 2010, in which the animals were intensively purchased. Most of the cattle herds in this region were eliminated or they introduced BVDV eradication by vaccinating animals and removing PIs. However these herds were excluded from our study. An analysis of BVDV in the selected geographical regions of Poland is shown in Table 2.

The statistical significance of the selected parameters considered to be a “risk” associated with BVDV infection is shown in Table 3. Only geographical location, access to pasture, and the purchases of new animals were statistically significant. All other analyzed parameters were not, including the presence of BVDV within herds. 

Data regarding on the productivity of tested herds (Table 4) shows that the selected parameters were not statistically significant or of direct importance for maintaining BVDV within herds. Thus, it can suggest that the long-term presence of infections might cause the specific “homeostasis” within herds, leading to BVDV infection being less noticeable to owners. Clear differences were only detected for the length of the period between calving (*p* = 0.009).

## 4. Discussion

The circulation of BVDV in cattle herds causes acute, subclinical, and persistent infections in animals. All three of the forms causing the described forms of infection generate health problems in animals and lead to the presence of persistently or temporarily infected animals [18]. This, phenomenon facilitates the circulation of the virus in the cattle population, regardless of the presence of BVDV antibodies in high level [19,20]. 

The presence of antibodies specific to BVDV is the first method used to detect the presence of PI animals and is the first step for diagnoses of the herds and controlling the spread of this virus in the region without the use vaccination [21,22]. Subsequently, other methods are used to confirm this, and the pursuit of further studies is used to identify this and to remove such infected animals as soon as possible [23]. It is important for control BVDV because this infection is considered to be distributed worldwide in farm animals, and evidence for the natural susceptibility of wildlife species [24,25] comes mainly from serological surveys.

Differences in BVDV prevalence among regions or the introduction of the virus into the herds previously free of BVDV are often associated with particular epidemiological determinants, including the density of cattle populations, the condition and age of the animals, animal trade, and pasturing practices [26]. This relationship was observed in Poland, where the seroprevalence in bulls at artificial insemination centers was 0.9% [27], whereas in eastern and south-eastern regions of Poland the seroprevalence was 3.85% in young calves between 6 and 12 months old [28]. On the other hand, the herd-level seroprevalence was 70.7% in herds in eighteen provinces in Poland. High levels of antibodies (COD values above 0.55) were found in 52.8% of positive herds [29]. There is not much information about the BVDV situation in the neighboring countries. In Estonia, approximately 70% of all dairy cows were investigated. The BVDV infection status was established in 315–350 herds during three sampling periods: 1993–1995, 1997–1998, 1999–2000. The prevalence of 46%, 16%, and 18% (respectively) for suspect PI herds was observed [30]. Specific BVDV-antibodies were detected in 713 of 1059 (67.3%) analyzed samples from cattle farms in Eastern Ukraine [31]. In Slovakia, the prevalence of BVDV was 69% in cattle between 6 and 12 months of age [32]. In Lower Saxony, in 1996, the seroprevalence was 45.3% in cattle up to 3 years of age [33], whereas in Bavaria, in 2013, about 78% of investigated cattle herds showed antibodies against BVDV [34]. In Lithuania, 29.9% of the herds were not infected with BVDV, and in 32.7% of the herds, between 70% and 100% of cattle were seropositive to BVDV in 1997–2001 [35]. The true prevalence of the basis of the criteria, such as BVDV-specific antibodies and antigens, based on various criteria in a cross-sectional study of 773 Belgian herds, was 47.4% and 4.4%, respectively. Overall, 83.4% of the farmers stated that typical problems associated with BVDV were absent [36]. Moreover, only 8.4% of all farmers who completed the questionnaire reported problems possibly related to BVDV in the previous three years. Large herds were significantly more likely to be BVDV seropositive (OR = 1.004; *p* < 0.01). In non-vaccinated herds, the detection of PI animals was significantly associated with BVDV seropositivity (OR = 13.8; *p* < 0.01). Herds that had BVDV-related problems in the previous three years were more likely to be BVDV seropositive (OR = 1.9; *p* < 0.05). This phenomenon only became non-significant (OR = 1.8; *p* = 0.08) when taking only a subset of herds that had not been vaccinating animals <12 months. The results of the present study have confirmed an active circulation of BVDV in a considerable number of Belgian cattle herds [36]. In the Asturias region of Spain, another region of Europe, seropositivity was 21%, and, at the same time, no PI animals were detected, however, the authors detected two major factors associated with seropositivity: age and cow origin. These studies indicate that BVDV infection could be controlled in the selected area by controlling livestock-trade (without vaccines) [37].

Several studies have evaluated the presence of BVDV infection in cattle at the individual and herd levels following the implementation of the eradication programs, which were assessed several times [38]. The analysis of potential risk factors was evaluated to identify which risks were associated with the appearance of virus-positive newborn calves in herds, where BVDV had not been previously detected. The identification of such “risk” factors would facilitate a targeted approach in the future. The evaluated factors included: herd size, early death rate (i.e., the number of animals that either die before 15 days of age or were stillborn per number of new born calves each per year), buying in stock, using communal summer grazing, production type, age structure, and management strategy [22]. In Scotland dairy farms noted the highest concentration of antibodies with a prevalence of 73% at the herd level [39]. Risk factor analysis suggested that routine vaccination against BVDV, the suspicion of BVD, the housing of pregnant cows with calves, the total number of cows, and the proportion of cows that were dry were all associated with the presence of more of anti-BVDV antibodies in bulk tank milk. The inclusion of BVD within the health plan for cattle in farms was associated with a decline in BVDV antibody levels in the BTM. Denmark was at the greatest risk of BVDV and the prevalence being associated with was related to the import of live cattle (with a median of 5.0%). Hoof trimming activity and the impact of animals imported from abroad represent the second most important introduction pathway (with a median of 2.4%). The risk of BVDV introduction due to imported semen, embryos, and visiting trucks visits was 0.4%, 1.0%, and 0.04%, respectively [40].

To identify the risk factors for BVDV infection in 300 randomly selected dairy herds in Brazil, BTM and indirect ELISA were used. Forty-three percent of herds were seropositive with artificial insemination and herd size being significantly associated with BVDV serological status (*p* < 0.05). The important risk factors were artificial insemination and an increasing number of visitors to the farm, who could introduce the BVDV virus through the contaminated clothes, shoes, and equipment [41]. In the cattle herds of Argentina, risk factors associated with BVDV included reproductive problems, such as outbreaks of abortions and calves with congenital defects. The BVDV appeared to be circulated via semen, resulting in PI infected animals becoming established. The average negative and positive rates of BVDV were 32.03% and 36.78%, respectively. Variable importance analysis showed that the important predictors of BVDV occurrence were: (a) who inseminates the animals, (b) the number of neighbouring farms that have cattle, and (c) the routine performance of rectal palpation [42]. The circulation of BVDV within herds generates two important problems: the generation of PI animals and economic losses associated with decreases in the fertility and reproductive efficiency. Consequently, the inability of PI animals to overcome normal bovine pathogens could result in clinical presentations more closely linked to the secondary pathogens than to the BVDV infection [43]. Our previous study, which was performed on 270 cattle herds between 2007 and 2009, showed that 53.9% of cattle had antibodies to BVDV. An analysis of the clinical signs of bovine viral diarrhea (BVD) commonly observed in dairy cattle led to the conclusion that sudden drops in milk yield (OR = 2.037), diarrhea in calves (OR = 1.422), and emaciation in juvenile (OR = 1.774) and adult animals (OR = 1.715) were more frequent in infected herds compared to uninfected herds. No other clinical signs considered as typical for infected herds increased, including spontaneous abortions and fetopathies, nor were any respiratory and/or alimentary tract disorders noted [17]. Luzzago et al. [44], in the northern Italy, classified dairy herds according BVDV serological status and the risk levels for factors related to the within-herd spread of BVDV introduction, such as: through livestock trade, the attendance of animals at shows/exhibitions, a common grazing pasture, the within-herd spread of BVDV, and the results of initial serological testing. The calculated odds ratios were significant for all categories, except for livestock trade. Thus, screening tests, the questionnaires, and the related risk assessments proved a practical approach for predicting the BVDV status of cattle herds.

The estimates of economic losses due to BVDV infection vary depending on the immunity status of a given population and the pathogenicity of the virus strains. The introduction of the infection into a totally susceptible population invariably causes extensive losses in cattle populations until a state of equilibrium is reached [21]. Infection with highly virulent BVDV strains, which cause severe clinical signs and death after the acute course of the disease, gives rise to substantial economic losses [10,20].

Although the impact of BVDV in the herds are difficult to identify, regular vaccination with the elimination of PI animals and the identification of risk factors for this population is a regular feature of eradication programs. In turn, for countries that have eliminated BVDV from the population, determining risk factors is all the more important because the circulation control of this virus is based only on monitoring studies, hence determining risk groups and comparing them between countries is particularly important.

## 5. Conclusions

The current study demonstrates that the rate of BVDV infection was most strongly correlated with the geographical location of the examined cattle populations in Poland. The second most significant factor was considered to be the grazing of animals and linked access to the pasture. The purchasing of new animals to be introduced to herds was also statistically significant. Confirmed “risk” factors were similar or identical and were dependent on the size of the stock, management practices, and the implementation of the eradication programs.

## Figures and Tables

**Table 1:** Presence of antibodies against the bovine viral diarrhea virus (BVDV) in examined dairy cattle herds, including the number and percentage of positive herds as well as the relative value of specific antibodies (s/p).

S/P value	Number of BTM Samples (n)	Results (%)	
val ≤ 20%	236	66.7	negative
20% < val ≤ 40%	36	10.2	positive
40% < val ≤ 60%	38	10.7	positive
60% < val ≤ 80%	28	7.9	positive
80% < val ≤ 100%	13	3.7	positive
100% < val	3	0.8	positive
Total	354	100	-

**Table 2:** Distribution of BVDV-positive herds in geographical regions of Poland.

Region	Voivodship	No of Herds (n)	No of Positive Herds (n)	Herd-level Seroprevalence (%)	CI 95%
C	Mazovia (n = 21) Łódź (n = 20)	41	16	39.0	25.7%, 54.3%
S	Lower Silesia (n = 17) Opole (n = 27) Silesia (n = 12) Lesser Poland (n = 3)	59	20	33.9	23.1%, 46.6%
N-W	Greater Poland (n = 93) Lubsz (n = 3) West-Pomerania (n = 2)	98	15	15.3	9.5%, 46.6%
N	Warmia-Masuria (n = 33) Kuyavia-Pomerania (n = 54) Pomerania (n = 6)	93	43	46.2	36.5%, 56.3%
E	Holy Cross Province (n = 2) Subcarpathia (n = 5) Lublin (n = 4) Podlaskie (n = 52)	63	24	38.1	27.1%, 50.4%
Total		354	118	33.3%	28.6%, 38.4%

**Table 3:** Estimation of the factors considered to be a “risk” for the presence of BVDV at the herd level.

Risk Factor	Category	n (%) of BVDV-Positive Herds in a Category	*p*-Value
Region of Poland	C	16/41 (39.0)	0.001
	S	20/59 (33.9)	
	N-W	15/98 (15.3)	
	N	43/93 (46.2)	
	E	24/63 (38.1)	
Average no of cows per 100ha	BVDV-positive: median of 17 (IQR from 16.3 to 21.5) cows/100ha		0.294
	BVDV-negative: median of 17 (IQR from 16.3 to 19.7) cows/100ha		
Herd size (n = 354)	≤50	22/44 (50.0)	0.506
	51–100	44/162 (27.2)	
	101–150	24/70 (34.3)	
	151–200	21/39 (53.9)	
	>200	7/39 (17.9)	
Number of cows in lactation (n = 349)	≤50	67/214 (31.3)	0.822
	51–100	39/99 (39.4)	
	>100	8/36 (22.2)	
Milk yield (n = 334)	≤5.0	5/11 (45.5)	0.31
	5.1–8.0	57/196 (29.1)	
	>8.0	47/127 (37.0)	
Pasture (n = 354)	Yes	35/143 (24.5)	0.004
	No	83/211 (39.3)	
Animals grazed (n = 143)	Calves	8/77 (10.4%)	<0.001
	Cows	16/36 (44.4%)	
	Both	11/30 (36.7%)	
Purchase of animals for replacement (n = 354)	Yes	46/176 (26.1)	0.004
	No	72/178 (40.5)	

**Table 4:** Production and clinical outcomes potentially linked to the BVD infection at the herd level.

Clinical Manifestation (n)	Category	n (%) of BVDV-Positive Herds in a Category	*p*-Value
First calving [month of life] (n = 354)	<24	9/22 (40.9)	0.814
	24–30	103/317 (32.5)	
	>30	6/15 (40.0)	
Average no of lactations per a cow (n = 328)	<2.4	14/42 (33.3)	0.949
	2.5–2.9	27/83 (32.5)	
	3.0–3.4	23/85 (27.1)	
	3.5–3.9	39/118 (33.1)	
Period between calving [days] (n = 354)	<380	15/40 (37.5)	0.009
	381–400	62/160 (38.7)	
	401–420	35/111 (31.5)	
	>420	6/43 (14.0)	
Foetopathy (n = 354)	Yes	10/30 (33.3)	0.999
	No	108/324 (33.3)	
Diarrhoea (n = 354)	Yes	44/114 (38.6)	0.148
	No	74/240 (30.8)	
Abortion (n = 354)	Yes	48/138 (34.8)	0.643
	No	70/216 (32.4)	
Problems with conception (n = 333)	very rare	23/44 (52.3)	0.301
	rare	18/48 (37.5)	
	moderately	20/97 (20.6)	
	often	20/76 (26.3)	
	very often	27/68 (39.7)	
Respiratory problems (n = 354)	Yes	41/133 (30.8)	0.438
	No	77/221 (34.8)	
Mastitis (n = 326)	very rare	20/49 (40.8)	0.34
	rare	22/119 (18.5)	
	moderately	31/71 (43.7)	
	often	13/47 (27.7)	
	very often	15/40 (37.5)	
Percent of cows with mastitis in last 2 months (n = 339)	0	10/27 (37.0)	0.506
	1–3	48/175 (27.4)	
	4–6	34/71 (47.9)	
	7–9	5/15 (33.3)	
	10–12	11/40 (27.5)	
	>12	2/11 (18.2)	
SCC ^1^ in the last month (n = 340)	<250	41/103 (39.8)	0.068
	251–350	37/108 (34.3)	
	351–400	15/93 (16.1)	
	401–500	11/21 (52.4)	
	>500	6/15 (40.0)	
SCC in the previous month (n = 332)	<250	36/98 (36.7)	0.186
	251–350	39/107 (36.5)	
	351–400	15/85 (17.7)	
	401–500	12/22 (54.5)	
	>500	6/20 (30.0)	
Lameness (n = 320)	very rare	34/83 (41.0)	0.164
	rare	14/51 (27.5)	
	moderately	21/59 (35.6)	
	often	21/99 (21.2)	
	very often	13/28 (46.4)	

^1^ Somatic Cell Counts.
